# Baicalein Alleviates Testicular Ischemia-Reperfusion Injury in a Rat Model of Testicular Torsion-Detorsion

**DOI:** 10.1155/2022/1603469

**Published:** 2022-11-04

**Authors:** Si-Ming Wei, Yu-Min Huang

**Affiliations:** ^1^Shulan International Medical College, Zhejiang Shuren University, Hangzhou City, Zhejiang Province 310015, China; ^2^School of Nursing, Zhejiang Chinese Medical University, Hangzhou City, Zhejiang Province 310053, China; ^3^Department of Sport Science, College of Education, Zhejiang University, Hangzhou City, Zhejiang Province 310058, China

## Abstract

Testicular torsion/detorsion-induced ischemia/reperfusion injury is partly due to the overgeneration of reactive oxygen species. Baicalein, a main bioactive constituent derived from the dried root of *Scutellaria baicalensis* Georgi, possesses powerful antioxidative and anti-inflammatory properties. Therefore, we designed the research to explore the possible protective effect of baicalein against testicular ischemia-reperfusion injury. Sprague-Dawley rats were randomized into 4 groups, including control, testicular ischemia-reperfusion, testicular ischemia-reperfusion+vehicle injection, and testicular ischemia-reperfusion+baicalein therapy groups. The control group received surgical exposure of the left testis without torsion-detorsion. In the testicular ischemia-reperfusion group, the left testis underwent 720° counterclockwise torsion for two hours and then was allowed detorsion. Rats in the testicular ischemia-reperfusion+vehicle injection group received intraperitoneal injection of the vehicle at detorsion. In the baicalein-treated group, the intraperitoneal administration of baicalein dissolved in the vehicle was performed at detorsion. At four hours or three months following testicular detorsion, testicular tissues were removed to detect the levels of tumor necrosis factor-alpha (TNF-*α*) and interleukin-1beta (IL-1*β*) which can recruit neutrophils into the testis, myeloperoxidase activity (an index of neutrophil infiltration in the testis), protein expression of nicotinamide adenine dinucleotide phosphate (NADPH) oxidase in neutrophils which can catalyze reactive oxygen species production, malondialdehyde concentration (a common marker of reactive oxygen species), and spermatogenesis. Both testicular ischemia-reperfusion and testicular ischemia-reperfusion+vehicle injection significantly increased the TNF-*α* and IL-1*β* levels, myeloperoxidase activity, NADPH oxidase protein expression, and malondialdehyde concentration, while decreased spermatogenesis in ipsilateral testes. In contrast, baicalein administration remarkably reduced TNF-*α* and IL-1*β* levels, myeloperoxidase activity, NADPH oxidase protein expression, and malondialdehyde concentration and also elevated spermatogenesis in ipsilateral testes. The results of our experiment demonstrate that baicalein alleviates testicular ischemia-reperfusion injury by inhibiting TNF-*α* and IL-1*β* secretion, neutrophil infiltration in the testis, and NADPH oxidase protein expression in neutrophils to reduce reactive oxygen species production.

## 1. Introduction

Testicular torsion caused by rotation of the spermatic cord around itself impedes testicular blood flow. Prolonged ischemic duration of the testis can result in testicular necrosis. The most common treatment method for testicular torsion is early surgical detorsion. It recovers testicular blood circulation and prevents ischemic necrosis. However, despite timely detorsion, testicular atrophy still occurs in 9.1%-73.3% of patients in the long-term follow-up [1, 2]. Damage caused by testicular torsion-detorsion is an ischemia-reperfusion injury. In the process of ischemia-reperfusion, excessive reactive oxygen species are formed in tissue [3–5]. Reactive oxygen species, like superoxide anions, nitric oxide, hydrogen peroxide, hypochlorous acid, hydroxyl radicals, and singlet oxygen, are highly reactive and can damage cellular constituents, such as lipids, proteins, deoxyribonucleic acid, and carbohydrates, finally leading to the loss of cellular viability and even cellular death [6]. Testicular tissue is very sensitive to reactive oxygen species because it contains a high amount of polyunsaturated fatty acids [7].

Currently, no effective drug for alleviating testicular ischemia-reperfusion injury is clinically available. *Scutellaria baicalensis* Georgi, also called Huangqin in China, is a perennial herb from the Lamiaceae family [8, 9]. It is indigenous to North China, Japan, North Korea, Mongolia, and Russia's Eastern Siberia and is widely cultivated in many European countries [9]. In China, *Scutellaria baicalensis* Georgi is a common Chinese medicinal herb and has been widely used for more than 2000 years to treat various diseases, such as diarrhea, dysentery, hypertension, hemorrhaging, insomnia, inflammation, and respiratory infections [9, 10]. Nowadays, it is officially listed in Chinese Pharmacopoeia, European Pharmacopoeia, and British Pharmacopoeia [9]. Baicalein (5,6,7-trihydroxyflavone) is a main bioactive constituent derived from the dried root of *Scutellaria baicalensis* Georgi [11]. Its molecular formula and molecular weight are C_15_H_10_O_5_ and 270.24, respectively [12]. Various studies have shown that baicalein possesses numerous bioactivities, including antioxidative, anti-inflammatory, antibacterial, antiviral, anticancer, antithrombotic, anxiolytic, antidepressant, and antifibrotic functions [13–21]. Furthermore, baicalein has been reported to ameliorate ischemia-reperfusion injury in the brain, liver, heart, kidney, spinal cord, and retina [22–27]. Nevertheless, whether or not baicalein mitigates testicular ischemia-reperfusion injury was not documented. Hence, in the present research, the effect of baicalein on ischemia-reperfusion injury induced by rat testicular torsion-detorsion was checked.

## 2. Materials and Methods

### 2.1. Laboratory Animals

In this study, male Sprague-Dawley rats were supplied by SLAC Laboratory Animal Co., Ltd., Shanghai City, China. A total of eighty male Sprague-Dawley rats (weight range of 250-300 g, 8 weeks old) were used for the experiment. They were kept in a standard animal house where humidity of 55% ± 5%, photoperiod of 12-hour light-dark cycles, and temperature of 21°C ± 1°C were maintained. Rats could unrestrictedly obtain sterilized pellet diet and autoclaved water. Before surgical procedure, rats stayed in the animal house for 7 days to adapt to the conditions. The institutional animal ethics committee of our university approved the experimental protocol. All animal procedures were conducted in the light of regulations of laboratory animal care and use.

### 2.2. Testicular Ischemia-Reperfusion Model

According to the study design, experimental animals were randomized into 4 groups (*n* = 20 in each group): control, testicular ischemia-reperfusion, testicular ischemia-reperfusion+vehicle injection, and testicular ischemia-reperfusion+baicalein therapy groups. Before surgical operation, ketamine (50 mg/kg; Sigma Chemical Company, St. Louis, MO, USA) was administered intraperitoneally to induce anesthesia. After clipping the hairs on the left-sided ilioinguinal region, the region was disinfected with 10% povidone-iodine solution. Subsequently, a left-sided ilioinguinal incision was performed, through which the left testis was pulled out. In the control group, we placed an atraumatic 11-0 suture through the tunica albuginea. Then, the left testis was returned to scrotal position without any other treatment, and the incision was stitched by a 4-0 nonabsorbable silk suture. The performance of testicular ischemia-reperfusion model was as follows. In order to induce testicular ischemia, a 720-degree rotation in a counterclockwise direction was performed on the left testis. To maintain the left testis in this state for 2 hours, the tunica albuginea of the testis and inner wall of the scrotum were stitched together with an atraumatic 11-0 suture. At the end of the testicular ischemic period, we removed the suture and restored the twisted testis to its original position for reperfusion. Baicalein (Sigma Chemical Company) was dissolved in a solution of dimethyl sulfoxide and promptly diluted to the final concentration of 10 mg/ml with normal saline before injection. The final concentration for dimethyl sulfoxide in the injected solution was 10% (*v*/*v*). At onset of reperfusion, rats in the testicular ischemia-reperfusion+baicalein therapy group received intraperitoneal injection of baicalein (100 mg/kg) dissolved in a solution of dimethyl sulfoxide and normal saline. The dosage of baicalein used in the current study was based on the results of several previous experimental researches [22, 26, 28–32]. Rats in the testicular ischemia-reperfusion+vehicle injection group underwent intraperitoneal injection of an equal volume of the vehicle (a solution of dimethyl sulfoxide and normal saline) at onset of reperfusion. Lastly, the reperfused testis was replaced into the scrotum, and the incision was stitched. As mentioned above, a total of 80 male rats were randomized into 4 groups (*n* = 20 in each group). After 4 hours of reperfusion, bilateral orchiectomy was performed on 10 rats in each group to examine tumor necrosis factor-alpha (TNF-*α*) and interleukin-1beta (IL-1*β*) levels, myeloperoxidase activity, nicotinamide adenine dinucleotide phosphate (NADPH) oxidase protein expression, and malondialdehyde content. Afterwards, these rats were euthanized via inhaling carbon dioxide. After 3 months of reperfusion, bilateral orchiectomy was performed on the remaining 10 rats in each group to measure testicular spermatogenesis.

### 2.3. Measurement of TNF-*α* and IL-1*β* Levels by Enzyme-Linked Immunosorbent Assay (ELISA)

Testicular sample (100 mg) was homogenized in ice-cold cell extraction buffer (Thermo Fisher Scientific, Waltham, MA, USA) with protease inhibitors (Sigma Chemical Company). After homogenization, the homogenate was centrifuged at 14,000 × gravity at 4°C for 15 minutes to obtain supernatant. The TNF-*α* and IL-1*β* levels in the supernatant were determined by using commercially available ELISA kits (PeproTech, Cranbury, NJ, USA) according to the manufacturer's instructions. The results of TNF-*α* and IL-1*β* levels were expressed as pg/mg tissue.

### 2.4. Determination of Myeloperoxidase Activity in the Testis

The tissue sample was placed in 50 mM potassium phosphate buffer, homogenized for 10 minutes, and centrifuged at 40,000 × gravity for 30 minutes. The pellet was obtained and suspended in 50 mM potassium phosphate buffer complemented with 0.5% hexadecyltrimethylammonium bromide (Sigma Chemical Company). The suspension was sonicated for 10 seconds and then subjected to 3 freeze-thaw cycles, after which the sonication was performed again for 10 seconds. Subsequently, the suspension was centrifuged again at 40,000 × gravity for 30 minutes, and supernatant was retained. Supernatant (100 *μ*l) was added to the reaction mixture containing 0.167 mg/ml O-dianisidine dihydrochloride (Sigma Chemical Company) and 0.0005% hydrogen peroxide. The change in absorbance per minute in each sample was measured at 460 nm by the use of a spectrophotometer. One unit activity of myeloperoxidase is defined as a quantity inducing degradation of 1 mM of peroxide per minute. Results were shown as U/g tissue.

### 2.5. Immunoblot for NADPH Oxidase

To detect NADPH oxidase protein expression, we homogenized testicular tissue in cold lysis solution (consisting of 50 mM Tris HCl, pH 7.4, 0.5% sodium deoxycholate, 1 mM dithiothreitol, 0.5 mM ethylenediaminetetraacetic acid, 0.5 *μ*g/ml leupeptin, 0.1% sodium dodecyl sulfate, 1 mM phenylmethylsulfonyl fluoride, 5 *μ*g/ml aprotinin, 150 mM NaCl, 2 mM sodium orthovanadate, and 1% nonidet P-40) to produce 10% (*w*/*v*) homogenate. Then, the homogenate was placed on ice for 30 minutes, and the supernatant was subsequently collected by centrifugation (14,000 *g*, 4°C) for 15 minutes. The protein concentration of each sample was measured by a Bradford assay kit (Bio-Rad Laboratories, Hercules, CA, USA). The protein sample was added to a loading buffer and heated at 100°C for 3 minutes. Equivalent protein samples (30 *μ*g/well) were electrophoresed on sodium dodecyl sulfate-polyacrylamide gel and then transferred electrophoretically to nitrocellulose membrane. The membrane was incubated in Tris-buffered saline containing 0.1% Tween-20 and 5% nonfat dry milk at room temperature for 1 hour to block nonspecific binding sites. Next, the membrane was probed overnight at 4°C with primary antibodies for anti-NADPH oxidase subunit gp91-phox (Santa Cruz Biotechnology, Santa Cruz, CA, USA) and anti-*β*-actin (inner control; Sigma Chemical Company). After being rinsed three times with Tris-buffered saline containing 0.1% Tween-20, the membrane was probed for 1 hour at room temperature with horseradish peroxidase-conjugated second antibody (Santa Cruz Biotechnology). Finally, the membrane was washed again three times in Tris-buffered saline containing 0.1% Tween-20, and antibody-reactive protein bands on the membrane were detected with enhanced chemiluminescence developer (Santa Cruz Biotechnology). A GS-700 imaging densitometer (Bio-Rad Laboratories) was used to analyze the intensity of NADPH oxidase and *β*-actin protein bands. The intensity ratio of NADPH oxidase band to internal reference *β*-actin band from the same sample expressed a relative level of NADPH oxidase protein expression.

### 2.6. Assessment of Testicular Malondialdehyde Concentration

Approximately 100 mg of testicular tissue sample was immersed in 1 ml chilled malondialdehyde lysis buffer. A homogenizer was used to homogenize the testicular tissue. The resulted suspension was centrifuged (5000 *g*, 15 minutes, 4°C), and the obtained supernatant was used for malondialdehyde analysis. Protein level of the supernatant was assessed using the method described by Bradford [33]. Testicular malondialdehyde concentration was determined using thiobarbituric acid reactive substance assay kit (Nanjing Jiancheng Institute of Bioengineering, Nanjing City, China) according to the method of Ohkawa et al. [34]. Results were shown as nmol/mg protein.

### 2.7. Spermatogenic Evaluation

Testicular spermatogenesis was evaluated by some markers, including testicular weight, seminiferous tubular diameter, number of germ cell layers, and Johnsen's score. Briefly, testicular specimen was weighed and placed into Bouin's solution for fixation. Thereafter, the sample was passed through the increased ethanol series and embedded in paraffin wax. The paraffin slice with 5 *μ*m thickness was obtained using a microtome. Section was deparaffinized, and then staining of section was performed using hematoxylin and eosin solutions (Sigma Chemical Company). Twenty round seminiferous tubules in each testicular section were inspected by a skilled pathologist in a blinded manner with the help of a light microscope. An eyepiece micrometer was installed on an optical microscope and used to determine seminiferous tubular diameter. Next, the number of germinal cell layers in the seminiferous tubule was assessed by counting the epithelial cell layer's number from basement membrane to tubular lumen. Moreover, the germinal epithelial cell maturity in the seminiferous tubule was evaluated by Johnsen scoring criteria, which range from 1 to 10 [35]. Score 10 represents normal spermatogenesis with a large number of spermatozoa, multilayered and organized germinal epithelium, and an open central lumen. Score 1 represents no cells in the seminiferous tubule.

### 2.8. Statistical Methods

Comparative analyses were conducted using the GraphPad Prism statistical software package, Version 4.0 (GraphPad Software Inc., San Diego, CA, USA). The values of all variables were shown as mean ± standard deviation. The Shapiro-Wilk test was used to confirm normal distribution of data. The differences in the variables between ipsilateral and contralateral testes within group were analyzed by a two-tailed Student *t*-test. One-way analysis of variance followed by Student-Newman-Keuls post hoc test was utilized to identify the differences in the data among four groups. The differences with *P* values less than 0.05 were regarded as statistically significant.

## 3. Results

### 3.1. TNF-*α* and IL-1*β* Levels in Testicular Tissue

**Figure 1:**
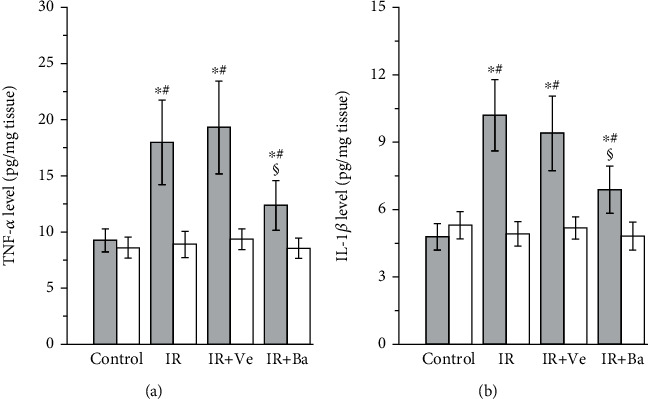
Effects of testicular ischemia-reperfusion (IR), testicular IR+vehicle (Ve) injection, and baicalein (Ba) treatment on (a) tumor necrosis factor-alpha (TNF-*α*) and (b) interleukin-1beta (IL-1*β*) levels in rat testicular tissue. Grey columns: ipsilateral testes; white columns: contralateral testes. Values are mean ± standard deviation (*n* = 10/group). ^∗^Statistically different from the control group (*P* < 0.05), ^#^statistically different from contralateral testes in the same group (*P* < 0.05), and ^§^statistically different from ipsilateral testes in the IR and IR+Ve groups (*P* < 0.05).

The results of TNF-*α* and IL-1*β* levels in the testes in control, testicular ischemia-reperfusion, testicular ischemia-reperfusion+vehicle injection, and testicular ischemia-reperfusion+baicalein therapy groups are shown in Figure 1. Compared with the control group, a significant increase was observed in TNF-*α* and IL-1*β* levels in ipsilateral torsional testes of the testicular ischemia-reperfusion and testicular ischemia-reperfusion+vehicle groups (*P* < 0.05). Treatment with baicalein caused a significant decrease in TNF-*α* and IL-1*β* levels of the ipsilateral testes, as compared with those in the testicular ischemia-reperfusion and testicular ischemia-reperfusion+vehicle groups (*P* < 0.05). No statistically significant differences were seen in the TNF-*α* and IL-1*β* levels in the contralateral nontorsional testes among four groups (*P* > 0.05).

### 3.2. Myeloperoxidase Activity in the Testis

**Figure 2:**
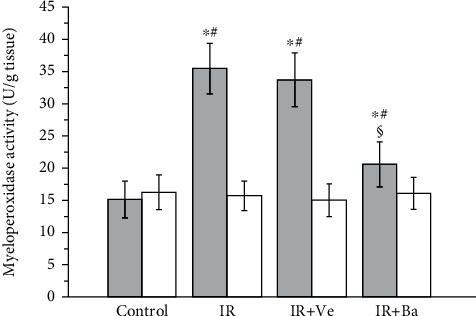
Effects of testicular ischemia-reperfusion (IR), testicular IR+vehicle (Ve) injection, and baicalein (Ba) treatment on myeloperoxidase activity in rat testicular tissue. Grey columns: ipsilateral testes; white columns: contralateral testes. Values are mean ± standard deviation (*n* = 10/group). ^∗^Statistically different from the control group (*P* < 0.05), ^#^statistically different from contralateral testes in the same group (*P* < 0.05), and ^§^statistically different from ipsilateral testes in the IR and IR+Ve groups (*P* < 0.05).

As shown in Figure 2, the myeloperoxidase activity in ipsilateral torsional testes in the testicular ischemia-reperfusion and testicular ischemia-reperfusion+vehicle groups was significantly higher compared with that in the control group (*P* < 0.05). However, baicalein treatment could significantly reduce myeloperoxidase activity of the ipsilateral testes in comparison with that in the testicular ischemia-reperfusion and testicular ischemia-reperfusion+vehicle groups (*P* < 0.05). There was no significant difference in myeloperoxidase activity of contralateral nontorsional testes among four groups (*P* > 0.05).

### 3.3. Testicular NADPH Oxidase Protein Expression

**Figure 3:**
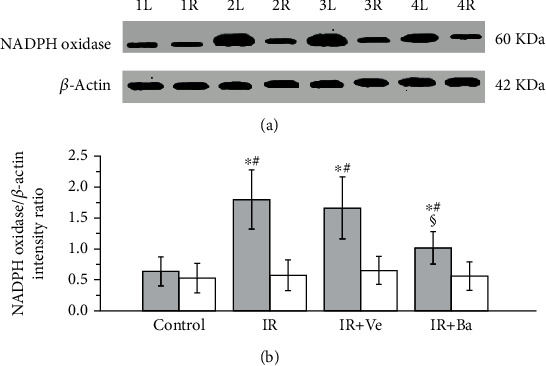
Effects of testicular ischemia-reperfusion (IR), testicular IR+vehicle (Ve) injection, and baicalein (Ba) treatment on nicotinamide adenine dinucleotide phosphate (NADPH) oxidase protein expression in rat testicular tissue. (a) Western blot analysis of NADPH oxidase protein expression in the testes of rats from the control, testicular IR, testicular IR+Ve injection, and Ba-treated groups. The *β*-actin protein expression in corresponding tissues is used as loading control. 1L and 1R: left (i.e., ipsilateral) and right (i.e., contralateral) testes in the control group; 2L and 2R: ipsilateral and contralateral testes in the testicular IR group; 3L and 3R: ipsilateral and contralateral testes in the testicular IR+Ve group; 4L and 4R: ipsilateral and contralateral testes in the Ba-treated group. (b) Histograms represent quantitative analysis of testicular NADPH oxidase protein expression in four groups. The intensity ratio of NADPH oxidase band to loading control *β*-actin band expresses a relative level of NADPH oxidase protein expression. Grey columns: ipsilateral testes; white columns: contralateral testes. Values are mean ± standard deviation (*n* = 10/group). ^∗^Statistically different from the control group (*P* < 0.05), ^#^statistically different from contralateral testes in the same group (*P* < 0.05), and ^§^statistically different from ipsilateral testes in the IR and IR+Ve groups (*P* < 0.05).

As shown in Figure 3, both testicular ischemia-reperfusion and testicular ischemia-reperfusion+vehicle injection significantly upregulated the NADPH oxidase protein expression in ipsilateral torsional testes in comparison with the control group (*P* < 0.05). Baicalein treatment significantly reduced the elevation of NADPH oxidase protein expression in ipsilateral testes triggered by both testicular ischemia-reperfusion and testicular ischemia-reperfusion+vehicle injection (*P* < 0.05). However, the NADPH oxidase protein expression in contralateral nontorsional testes was not considerably different among four groups (*P* > 0.05).

### 3.4. Testicular Concentration of Malondialdehyde

**Figure 4:**
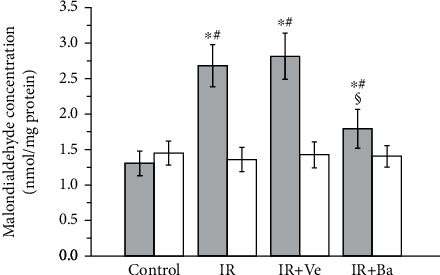
Effects of testicular ischemia-reperfusion (IR), testicular IR+vehicle (Ve) injection, and baicalein (Ba) treatment on malondialdehyde concentration in rat testicular tissue. Grey columns: ipsilateral testes; white columns: contralateral testes. Values are mean ± standard deviation (*n* = 10/group). ^∗^Statistically different from the control group (*P* < 0.05), ^#^statistically different from contralateral testes in the same group (*P* < 0.05), and ^§^statistically different from ipsilateral testes in the IR and IR+Ve groups (*P* < 0.05).

As shown in Figure 4, the malondialdehyde concentration in ipsilateral torsional testes was increased significantly following both testicular ischemia-reperfusion and testicular ischemia-reperfusion+vehicle injection in contrast to the control group (*P* < 0.05). After baicalein administration, the malondialdehyde concentration in ipsilateral testes was decreased significantly when compared with the testicular ischemia-reperfusion and testicular ischemia-reperfusion+vehicle groups (*P* < 0.05). Moreover, the statistical comparisons of malondialdehyde concentration in contralateral nontorsional testes revealed that there were no significant changes among four groups (*P* > 0.05).

### 3.5. Testicular Spermatogenic Findings

**Figure 5:**
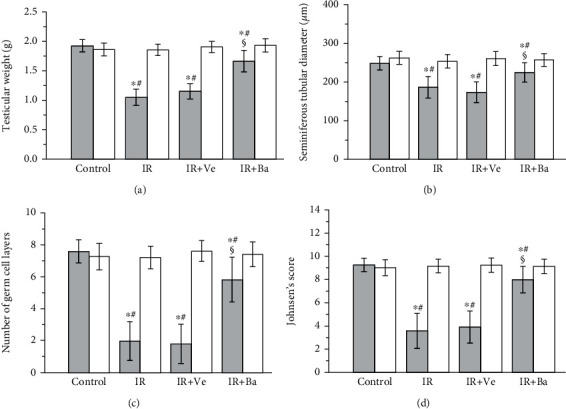
Effects of testicular ischemia-reperfusion (IR), testicular IR+vehicle (Ve) injection, and baicalein (Ba) treatment on (a) testicular weight, (b) seminiferous tubular diameter, (c) number of germ cell layers, and (d) Johnsen's score in rat testicular tissue. Grey columns: ipsilateral testes; white columns: contralateral testes. Values are mean ± standard deviation (*n* = 10/group). ^∗^Statistically different from the control group (*P* < 0.05), ^#^statistically different from contralateral testes in the same group (*P* < 0.05), and ^§^statistically different from ipsilateral testes in the IR and IR+Ve groups (*P* < 0.05).

**Figure 6:**
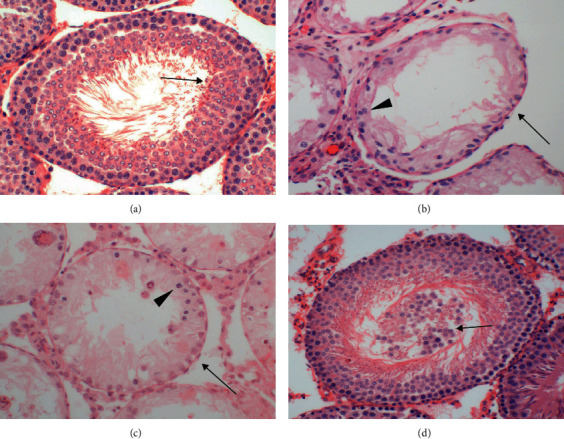
Histopathological features of testicular tissue in the control, testicular ischemia-reperfusion, testicular ischemia-reperfusion+vehicle injection, and baicalein-treated groups 3 months after operation (H&E staining, ×200). (a) Ipsilateral testes in the control group and contralateral testes in the four experimental groups all showed normal appearance in seminiferous tubular diameter and germinal cell layers. The germinal cells which matured to the level of spermatozoa (↑) were observed. In addition, an open lumen was present in the seminiferous tubule. Ipsilateral testes in the (b) testicular ischemia-reperfusion and (c) testicular ischemia-reperfusion+vehicle groups exhibited reduced testicular weight (Figure 5) and atrophic seminiferous tubules with reduced seminiferous tubular diameter (↑), disrupted germinal cell layers (▲), and spermatogenic arrest (▲). No spermatozoa were seen in atrophic seminiferous tubules. (d) Seminiferous tubules in ipsilateral testes of the baicalein-treated group were similar to those in the control group. Nevertheless, some germinal epithelial cells (↑) shed into the tubular lumen. These sloughed germinal epithelial cells could stop up the seminiferous tubule.

As shown in Figures 5 and 6, testicular weight, seminiferous tubular diameter, number of germ cell layers, and Johnsen's score were significantly lower in ipsilateral torsional testes in the testicular ischemia-reperfusion and testicular ischemia-reperfusion+vehicle groups than the control group (*P* < 0.05). In the baicalein-treated group, it was observed that the four parameters in ipsilateral testes had significantly improved compared with the testicular ischemia-reperfusion and testicular ischemia-reperfusion+vehicle groups (*P* < 0.05). In contrast, the differences in the four parameters of contralateral nontorsional testes were not significant among four groups (*P* > 0.05).

## 4. Discussion

Testicular torsion, also called spermatic cord torsion, is one of the most critical emergencies in the department of urology. Its annual incidence is 1 in 4000 males between 1 and 25 years of age [36]. Urgent surgical detorsion is an essential therapeutic approach in order to avoid testicular torsion-induced infarction. Survival will be achieved in 90%-100% of the affected testes if surgical detorsion is performed within 6 hours of the onset of acute testicular pain [37]. Testicular atrophy rates remain between 9.1% and 73.3% despite early surgical detorsion [1, 2]. In our study, 2-hour left testicular torsion, along with 3-month detorsion, caused damage to spermatogenesis in ipsilateral testes, which was reflected by significant decreases in testicular weight, seminiferous tubular diameter, number of germ cell layers, and Johnsen's score (Figures 5 and 6). Testicular damage that occurs during testicular torsion-detorsion is called as ischemia-reperfusion injury.

Ischemia-reperfusion injury is related to overgeneration of reactive oxygen species [3–5]. Increased reactive oxygen species have deleterious effects on cellular components by causing lipid peroxidation, structural alteration of protein, and DNA oxidation [6]. Owing to extremely reactive nature and short life of reactive oxygen species, their measurement is very difficult [38]. Reactive oxygen species have a peroxidative effect on cellular membrane lipids [6]. The final product of lipid peroxidation is malondialdehyde [38]. Hence, malondialdehyde is considered as a common marker of reactive oxygen species [38]. Our results revealed an increased malondialdehyde level and reduced spermatogenesis in ipsilateral testes of the testicular ischemia-reperfusion and testicular ischemia-reperfusion+vehicle groups, compared to the control group (Figures 4–6). These findings demonstrate that the production of excessive reactive oxygen species induces the loss of spermatogenesis during testicular ischemia-reperfusion. However, there were no significant differences in malondialdehyde level and spermatogenesis of ipsilateral testes between the testicular ischemia-reperfusion and testicular ischemia-reperfusion+vehicle groups (Figures 4–6). These data suggest that the vehicle has no effect on testicular ischemia-reperfusion injury. We also observed that malondialdehyde level was significantly decreased, while spermatogenesis was significantly increased in ipsilateral testes of the baicalein-treated group when compared to the testicular ischemia-reperfusion and testicular ischemia-reperfusion+vehicle groups (Figures 4–6). These results imply that baicalein exerts protective effect against testicular ischemia-reperfusion injury via decreasing the level of reactive oxygen species. Moreover, the clinical application of baicalein aluminum capsule has been found to be safe and efficacious in the treatment of enteritis and dysentery since it was put on the market in 1976, with effective rates of up to 97.27% for acute and chronic enteritis and 93% for bacillary dysentery [39]. Taken together, these data may provide a rationale for the clinical application of baicalein to treat testicular ischemia-reperfusion injury. Nevertheless, the mechanisms associated with baicalein-mediated reduction of reactive oxygen species are still unclear.

Inflammation is considered to contribute substantially to testicular ischemia-reperfusion injury [40]. During testicular ischemia-reperfusion, germ cells and interstitial macrophages in testis produce a large number of proinflammatory cytokines, such as TNF-*α* and IL-1*β* [41]. These cytokines induce chemotactic movement of neutrophils into ischemia-reperfusion testis [41]. A major source of reactive oxygen species overgeneration during tissular ischemia-reperfusion is neutrophils that infiltrate into ischemia-reperfusion tissue [42, 43]. NADPH oxidase in neutrophils plays an important role in reactive oxygen species generation [42, 44]. Calcium influx into neutrophils during ischemia activates NADPH oxidase [45]. During the reperfusion period, oxygen is reintroduced into ischemic tissue. Activated NADPH oxidase catalyzes the conversion of oxygen into superoxide anion [42]. The reaction of superoxide anion with itself produces hydrogen peroxide [46]. Superoxide anion reacts with hydrogen peroxide to generate hydroxyl radical [47]. Consequently, increased reactive oxygen species are formed in the process of ischemia-reperfusion. These increased reactive oxygen species are a reason for cellular injury [6]. Myeloperoxidase is an enzyme found primarily in azurophilic granules of neutrophils [48]. The enzyme activity reflects the number of neutrophils in the inflamed tissue [48]. Hence, myeloperoxidase activity serves as an index of neutrophil infiltration in tissue [48]. The glycoprotein gp91-phox is an essential component of the NADPH oxidase in neutrophils [49, 50]. For this reason, we used anti-NADPH oxidase subunit gp91-phox antibody to detect NADPH oxidase protein expression. In the present study, both testicular ischemia-reperfusion and testicular ischemia-reperfusion+vehicle injection caused significant increases in TNF-*α* and IL-1*β* levels, myeloperoxidase activity (an index of neutrophil infiltration in tissue), NADPH oxidase protein expression, and malondialdehyde content (a reliable index of reactive oxygen species) and resulted in a significant decrease in spermatogenesis in ipsilateral testes (Figures 1–6). These findings imply that testicular ischemia-reperfusion leads to excessive secretion of TNF-*α* and IL-1*β*, increased neutrophil recruitment into the testis, upregulation of NADPH oxidase expression in neutrophils, and reactive oxygen species overproduction, which can cause spermatogenic injury. Furthermore, our study indicated that baicalein treatment significantly reduced TNF-*α* and IL-1*β* levels, myeloperoxidase activity, NADPH oxidase expression, and malondialdehyde content and significantly improved spermatogenesis in ipsilateral testes (Figures 1–6). These data suggest that baicalein protects testicular spermatogenesis through the inhibition of TNF-*α* and IL-1*β* secretion, neutrophil recruitment into the testis, and NADPH oxidase protein expression in neutrophils to decrease reactive oxygen species production.

Baicalein at a dose of 100 mg/kg showed effective therapeutic effects on ischemia-reperfusion injury in murine liver, brain, and spinal cord [22, 26, 28–32]. Thus, we selected the dose in a murine model of testicular ischemia-reperfusion injury. In the current research, baicalein was only injected once at a single dose of 100 mg/kg body weight. Baicalein administration ameliorated histopathological damage in ipsilateral testes and recovered spermatogenesis to near normal (Figures 5 and 6). We did not investigate the therapeutic effect of baicalein at different administration doses or administration frequencies in this study. Hence, additional research is required to determine the most effective dose and frequency of baicalein administration.

The condition of the contralateral untwisted testis is debatable after unilateral testicular ischemia-reperfusion. In some studies, it has been reported that unilateral testicular ischemia-reperfusion induces harmful effect on the contralateral testis [51, 52]. In contrast, other studies have indicated that no injury is observed in the contralateral testis [53, 54]. Our results revealed that TNF-*α* and IL-1*β* levels, myeloperoxidase activity, NADPH oxidase expression, malondialdehyde content, and spermatogenesis of contralateral testes in the testicular ischemia-reperfusion group were not statistically different from those in the control group (Figures 1–6). These findings suggest that unilateral testicular ischemia-reperfusion does not affect the contralateral testis.

The testicular ischemia-reperfusion+vehicle injection group played an important role in our study. The results of comparison in spermatogenesis of ipsilateral testes between the testicular ischemia-reperfusion and testicular ischemia-reperfusion+vehicle groups showed that the vehicle had no effect on testicular ischemia-reperfusion injury (Figures 5 and 6). In addition, the results of comparison in spermatogenesis of ipsilateral testes between the testicular ischemia-reperfusion+baicalein dissolved in the vehicle and testicular ischemia-reperfusion+vehicle groups indicated that baicalein attenuated testicular ischemia-reperfusion injury (Figures 5 and 6). Consequently, the testicular ischemia-reperfusion+vehicle group is necessary to the present study.

Our study revealed that unilateral testicular ischemia-reperfusion led to ipsilateral testicular atrophy, which was characterized by reduced testicular weight and atrophic seminiferous tubules with reduced seminiferous tubular diameter, disrupted germinal cell layers, spermatogenic arrest, and absence of spermatozoa (Figures 5 and 6). The Masson trichrome-stained testicular section is often used to quantify collagen deposition as an index of fibrosis [55]. Some investigators used Masson trichrome-stained testicular section to find that testicular atrophy after testicular ischemia-reperfusion was usually accompanied by testicular fibrosis [55–58]. In a later study, we will try to use Masson trichrome-stained testicular section to show fibrosis in atrophic testes.

## 5. Conclusion

Our study is the first to indicate that baicalein exerts a protective effect against ischemia/reperfusion-induced testicular injury via inhibiting TNF-*α* and IL-1*β* secretion, neutrophil infiltration in the testis, and NADPH oxidase protein expression in neutrophils to reduce reactive oxygen species production. These results suggest that baicalein may be an effective therapeutic agent for patients with testicular torsion-detorsion. However, human trials should be conducted to validate the clinical benefit of baicalein.

## Data Availability

All data used to support the findings of this study are included in the article.

## References

[1] Moore S. L., Chebbout R., Cumberbatch M. (2021). Orchidopexy for testicular torsion: a systematic review of surgical technique. *European Urology Focus*.

[2] Zvizdic Z., Aganovic A., Milisic E., Jonuzi A., Zvizdic D., Vranic S. (2021). Duration of symptoms is the only predictor of testicular salvage following testicular torsion in children: a case-control study. *The American Journal of Emergency Medicine*.

[3] Liu H., Shi M., Li X. (2022). Adipose mesenchymal stromal cell-derived exosomes prevent testicular torsion injury via activating PI3K/AKT and MAPK/ERK1/2 pathways. *Oxidative Medicine and Cellular Longevity*.

[4] Semercioz A., Baltaci A. K., Mogulkoc R., Avunduk M. C. (2017). Effect of zinc and melatonin on oxidative stress and serum inhibin-B levels in a rat testicular torsion-detorsion model. *Biochemical Genetics*.

[5] Duman A., Mogulkoc R., Baltaci A. K., Menevse E. (2015). 3′, 4′-Dihydroxyflavonol attenuates tissue damage in unilateral testis ischemia-reperfusion in rats. *Bratislavské Lekárske Listy*.

[6] Di Mascio P., Martinez G. R., Miyamoto S., Ronsein G. E., Medeiros M. H. G., Cadet J. (2019). Singlet molecular oxygen reactions with nucleic acids, lipids, and proteins. *Chemical Reviews*.

[7] Agarwal A., Makker K., Sharma R. (2008). Review article: Clinical relevance of oxidative stress in male factor infertility: an update. *American Journal of Reproductive Immunology*.

[8] Zhao T., Tang H., Xie L. (2019). Scutellaria baicalensis Georgi. (Lamiaceae): a review of its traditional uses, botany, phytochemistry, pharmacology and toxicology. *The Journal of Pharmacy and Pharmacology*.

[9] Xiang L., Gao Y., Chen S., Sun J., Wu J., Meng X. (2022). Therapeutic potential of Scutellaria baicalensis Georgi in lung cancer therapy. *Phytomedicine*.

[10] Pei T., Yan M., Huang Y., Wei Y., Martin C., Zhao Q. (2022). Specific flavonoids and their biosynthetic pathway in Scutellaria baicalensis. *Frontiers in Plant Science*.

[11] Li L., Gao H., Lou K. (2021). Safety, tolerability, and pharmacokinetics of oral baicalein tablets in healthy Chinese subjects: a single-center, randomized, double-blind, placebo- controlled multiple-ascending-dose study. *Clinical and Translational Science*.

[12] Yin B., Li W., Qin H., Yun J., Sun X. (2021). The use of Chinese skullcap (Scutellaria baicalensis) and its extracts for sustainable animal production. *Animals (Basel)*.

[13] Choi E. O., Jeong J. W., Park C. (2016). Baicalein protects C6 glial cells against hydrogen peroxide-induced oxidative stress and apoptosis through regulation of the Nrf2 signaling pathway. *International Journal of Molecular Medicine*.

[14] Li J., Wang Y., Wu T., Li S., Sun Y. N., Liu Z. H. (2022). Baicalein suppresses high glucose-induced inflammation and apoptosis in trophoblasts by targeting the miRNA-17-5p-Mfn1/2-NF-*κ*B pathway. *Placenta*.

[15] Liu T., Luo J., Bi G., Du Z., Kong J., Chen Y. (2020). Antibacterial synergy between linezolid and baicalein against methicillin-resistant Staphylococcus aureus biofilm in vivo. *Microbial Pathogenesis*.

[16] Low Z. X., OuYong B. M., Hassandarvish P., Poh C. L., Ramanathan B. (2021). Antiviral activity of silymarin and baicalein against dengue virus. *Scientific Reports*.

[17] Zhang F. W., Peng L. Y., Shi C. J. (2022). Baicalein mediates the anti-tumor activity in osteosarcoma through lncRNA-NEF driven Wnt/*β*-catenin signaling regulatory axis. *Journal of Orthopaedic Translation*.

[18] Huang Y., Tsang S. Y., Yao X., Chen Z. Y. (2005). Biological properties of baicalein in cardiovascular system. *Current Drug Targets. Cardiovascular & Haematological Disorders*.

[19] de Carvalho R. S., Duarte F. S., de Lima T. C. (2011). Involvement of GABAergic non-benzodiazepine sites in the anxiolytic-like and sedative effects of the flavonoid baicalein in mice. *Behavioural Brain Research*.

[20] Xiong Z., Jiang B., Wu P. F. (2011). Antidepressant effects of a plant-derived flavonoid baicalein involving extracellular signal-regulated kinases cascade. *Biological & Pharmaceutical Bulletin*.

[21] Wang W., Zhou P. H., Xu C. G., Zhou X. J., Hu W., Zhang J. (2016). Baicalein ameliorates renal interstitial fibrosis by inducing myofibroblast apoptosis in vivo and in vitro. *BJU International*.

[22] Li S., Zhang Y., Fei L. (2022). Baicalein-ameliorated cerebral ischemia-reperfusion injury dependent on calpain 1/AIF pathway. *Bioscience, Biotechnology, and Biochemistry*.

[23] Drefs M., Thomas M. N., Guba M. (2017). Modulation of glutathione hemostasis by inhibition of 12/15-lipoxygenase prevents ROS-mediated cell death after hepatic ischemia and reperfusion. *Oxidative Medicine and Cellular Longevity*.

[24] Song L., Yang H., Wang H. X. (2014). Inhibition of 12/15 lipoxygenase by baicalein reduces myocardial ischemia/reperfusion injury via modulation of multiple signaling pathways. *Apoptosis*.

[25] Wu K., Li H., Tian J., Lei W. (2015). Protective effect of baicalein on renal ischemia/reperfusion injury in the rat. *Renal Failure*.

[26] Wu C., Xu H., Li J. (2020). Baicalein attenuates pyroptosis and endoplasmic reticulum stress following spinal cord ischemia-reperfusion injury via autophagy enhancement. *Frontiers in Pharmacology*.

[27] Pan L., Sze Y. H., Yang M. (2022). Baicalein-a potent pro-homeostatic regulator of microglia in retinal ischemic injury. *Frontiers in Immunology*.

[28] Liu A., Huang L., Guo E. (2016). Baicalein pretreatment reduces liver ischemia/reperfusion injury via induction of autophagy in rats. *Scientific Reports*.

[29] Zhou Y., Tan Z., Huang H. (2021). Baicalein pre-treatment alleviates hepatic ischemia/reperfusion injury in mice by regulating the Nrf2/ARE pathway. *Experimental and Therapeutic Medicine*.

[30] Liu A., Huang L., Fan H. (2015). Baicalein pretreatment protects against liver ischemia/reperfusion injury via inhibition of NF-*κ*B pathway in mice. *International Immunopharmacology*.

[31] Li W. H., Yang Y. L., Cheng X. (2020). Baicalein attenuates caspase-independent cells death via inhibiting PARP-1 activation and AIF nuclear translocation in cerebral ischemia/reperfusion rats. *Apoptosis*.

[32] Ran Y., Qie S., Gao F. (2021). Baicalein ameliorates ischemic brain damage through suppressing proinflammatory microglia polarization via inhibiting the TLR4/NF-*κ*B and STAT1 pathway. *Brain Research*.

[33] Bradford M. M. (1976). A rapid and sensitive method for the quantitation of microgram quantities of protein utilizing the principle of protein-dye binding. *Analytical Biochemistry*.

[34] Ohkawa H., Ohishi N., Yagi K. (1979). Assay for lipid peroxides in animal tissues by thiobarbituric acid reaction. *Analytical Biochemistry*.

[35] Johnsen S. G. (1970). Testicular biopsy score count--a method for registration of spermatogenesis in human testes: normal values and results in 335 hypogonadal males. *Hormones*.

[36] Yecies T., Bandari J., Schneck F., Cannon G. (2018). Direction of rotation in testicular torsion and identification of predictors of testicular salvage. *Urology*.

[37] Pogorelić Z., Mustapić K., Jukić M. (2016). Management of acute scrotum in children: a 25-year single center experience on 558 pediatric patients. *The Canadian Journal of Urology*.

[38] Kohsaka T., Yoneda Y., Yoshida T. (2022). Relaxin exerts a protective effect during ischemia-reperfusion in the rat model. *Andrology*.

[39] Liao H., Ye J., Gao L., Liu Y. (2021). The main bioactive compounds of Scutellaria baicalensis Georgi. for alleviation of inflammatory cytokines: a comprehensive review. *Biomedicine & Pharmacotherapy*.

[40] Lysiak J. J., Turner S. D., Nguyen Q. A., Singbartl K., Ley K., Turner T. T. (2001). Essential role of neutrophils in germ cell-specific apoptosis following ischemia/reperfusion injury of the mouse testis. *Biology of Reproduction*.

[41] Lysiak J. J. (2004). The role of tumor necrosis factor-alpha and interleukin-1 in the mammalian testis and their involvement in testicular torsion and autoimmune orchitis. *Reproductive Biology and Endocrinology*.

[42] Babior B. M., Peters W. A. (1981). The O_2_--producing enzyme of human neutrophils. Further properties. *Journal of Biological Chemistry*.

[43] Ahmed A. I., Lasheen N. N., El-Zawahry K. M. (2016). Ginkgo biloba ameliorates subfertility induced by testicular ischemia/reperfusion injury in adult Wistar rats: a possible new mitochondrial mechanism. *Oxidative Medicine and Cellular Longevity*.

[44] Bedard K., Krause K. H. (2007). The NOX family of ROS-generating NADPH oxidases: physiology and pathophysiology. *Physiological Reviews*.

[45] Irita K., Fujita I., Takeshige K., Minakami S., Yoshitake J. (1986). Calcium channel antagonist induced inhibition of superoxide production in human neutrophils: mechanisms independent of antagonizing calcium influx. *Biochemical Pharmacology*.

[46] Root R. K., Metcalf J. A. (1977). H_2_O_2_ release from human granulocytes during phagocytosis. Relationship to superoxide anion formation and cellular catabolism of H_2_O_2_: studies with normal and cytochalasin B-treated cells. *The Journal of Clinical Investigation*.

[47] Beauchamp C., Fridovich I. (1970). A mechanism for the production of ethylene from methional: the generation of the hydroxyl radical by xanthine oxidase. *The Journal of Biological Chemistry*.

[48] Bradley P. P., Priebat D. A., Christensen R. D., Rothstein G. (1982). Measurement of cutaneous inflammation: estimation of neutrophil content with an enzyme marker. *The Journal of Investigative Dermatology*.

[49] Cross A. R., Segal A. W. (2004). The NADPH oxidase of professional phagocytes--prototype of the NOX electron transport chain systems. *Biochimica et Biophysica Acta*.

[50] Quinn M. T., Gauss K. A. (2004). Structure and regulation of the neutrophil respiratory burst oxidase: comparison with nonphagocyte oxidases. *Journal of Leukocyte Biology*.

[51] Hu Z., Liu Q., Yan Z., Wang Q., Liu J. (2022). Protective effect of remote ischemic postconditioning in rat testes after testicular torsion/detorsion. *Andrology*.

[52] Akhigbe R. E., Hamed M. A., Odetayo A. F., Akhigbe T. M., Ajayi A. F., Ajibogun F. A. H. (2021). Omega-3 fatty acid rescues ischaemia/perfusion-induced testicular and sperm damage via modulation of lactate transport and xanthine oxidase/uric acid signaling. *Biomedicine & Pharmacotherapy*.

[53] Jafarova Demirkapu M., Karabag S., Akgul H. M., Mordeniz C., Yananli H. R. (2022). The effects of etomidate on testicular ischemia reperfusion injury in ipsilateral and contralateral testes of rats. *European Review for Medical and Pharmacological Sciences*.

[54] Quintaes I. P. P., de Avelar G. F., Quintaes A. P., Boasquevisque P. C. R., Resende V. (2020). Epithelial growth factor and decompressive testicular fasciotomy to control ischemia reperfusion injury in rats. *Journal of Pediatric Urology*.

[55] Ho C., Zee R. S., Omidi N. (2021). Varenicline limits ischemia reperfusion injury following testicular torsion in mice. *Journal of Pediatric Urology*.

[56] Aydın A., Sönmez M. G., Ecer G. (2021). The effect of intratesticular dexpanthenol on experimentally-induced testicular ischaemia/reperfusion injury. *Journal of Pediatric Urology*.

[57] Yildirim C., Yuksel O. H., Urkmez A., Sahin A., Somay A., Verit A. (2018). Protective effects of Tadalafil and darbepoetin against ischemia - reperfusion injury in a rat testicular torsion model. *International Braz J Urol*.

[58] Moreno D., Sobarzo C. M., Lustig L., Rodríguez Peña M. G., Guazzone V. A. (2020). Effect of ketotifen fumarate on experimental autoimmune orchitis and torsion of the spermatic cord. *Asian Journal of Andrology*.

